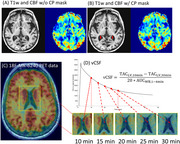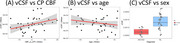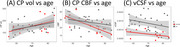# Choroid Plexus Blood Flow, Not Volume, Drives Cerebrospinal Fluid Turnover in Alzheimer's disease

**DOI:** 10.1002/alz70856_105139

**Published:** 2026-01-08

**Authors:** Liangdong Zhou, Xiuyuan Hugh Wang, Tracy A Butler, Gloria Chiang, Mony J. de Leon, Yi Li

**Affiliations:** ^1^ Weill Cornell Medicine, New York City, NY, USA

## Abstract

**Background:**

Cerebrospinal fluid (CSF) turnover plays a vital role in maintaining brain homeostasis and clearing neurotoxic substances, such as amyloid‐beta and tau proteins, which is implicated in Alzheimer's disease (AD) and mild cognitive impairment (MCI). The choroid plexus (CP), responsible for CSF production, has been hypothesized to influence CSF turnover via its blood flow and volume. However, the relative contributions of CP blood flow and volume to lateral ventricle CSF turnover, particularly in the context of aging and neurodegeneration, remain poorly understood.

**Method:**

We analyzed data from 40 subjects (age 69.92±8.56 years, 13 males), including 13 diagnosed with MCI/AD and 27 cognitively normal controls (CN), using multimodal imaging to measure lateral ventricle CSF turnover rate (vCSF)measured from dynamic 18F‐MK6240 PET and CP blood flow estimated using arterial spin labeling (ASL) magnetic resonance (MR). CP volume was assessed using MR T1w and T2FLAIR. See Figure 1. Multivariable linear regression models were employed to evaluate the associations of CSF turnover with diagnosis group, CP blood flow, CP volume, and age, adjusting for sex and intracranial volume (ICV). Additional analyses examined the relationships between these variables and age controlling for sex and diagnosis.

**Result:**

Lateral ventricle CSF turnover rate vCSF was significantly associated with diagnostic group (MCI/AD: t=‐4.27, *p* <0.001) and CP blood flow (t=2.11, *p* <0.05), but not CP volume (t=0.79, *p* = 0.43), see Figure 2. Both CSF turnover rate (t=‐1.93, *p* <0.05) and CP blood flow (t=‐2.72, *p* <0.05) were negatively associated with age. In contrast, CP volume exhibited a positive association with age (t=2.71, *p* <0.05). See Figure 3. These findings indicate that CP blood flow, rather than its volume, is a critical driver of CSF turnover in the lateral ventricle, particularly in the context of aging and neurodegeneration.

**Conclusion:**

Our results suggest that reduced CP blood flow, rather than structural changes in CP volume, underlies the decline in CSF turnover observed in aging and neurodegenerative conditions such as MCI/AD. These findings highlight the importance of maintaining CP vascular function for preserving CSF turnover and brain health in aging populations. Future studies should validate these findings in larger cohorts and explore causal links between CP perfusion, CSF turnover, and amyloid/tau accumulation.